# CMOS-Compatible AlScN Memristor on Silicon Exhibiting Short-Term Memory for Reservoir Computing

**DOI:** 10.3390/biomimetics11080519

**Published:** 2026-07-23

**Authors:** Woohyun Park, Hyojeong Chae, Maria Rasheed, Sungjun Kim

**Affiliations:** 1 Division of Electronics and Electrical Engineering, Dongguk University, Seoul 04620, Republic of Korea; 2 Department of Advanced Battery Convergence Engineering, Dongguk University, Seoul 04620, Republic of Korea

**Keywords:** ferroelectric, memristor, aluminum scandium nitride, short-term memory, neuromorphic, reservoir computing, in-memory computing

## Abstract

We report a CMOS-compatible ferroelectric memristor based on a TiN/AlScN/n^+^ Si metal ferroelectric semiconductor (MFS) structure, fabricated entirely via low-temperature sputtering processes. The ultrathin AlScN film exhibits robust ferroelectricity with a high remanent polarization (2P_r_ ≈ 80.91 μC/cm^2^) and excellent endurance over 10^5^ cycles, while maintaining uniform switching across cells. Notably, the use of a heavily doped silicon bottom electrode enables full compatibility with conventional back-end-of-line (BEOL) CMOS processes and facilitates integration with silicon-based circuits. Beyond stable memory performance, the device demonstrates volatile short-term memory (STM) behavior originating from depolarization field-induced polarization relaxation, which is essential for neuromorphic dynamics. Leveraging this STM feature, the device was implemented as a physical reservoir in a reservoir computing (RC) framework, achieving 97.64% classification accuracy on the MNIST dataset using temporally coded inputs. These results highlight the potential of AlScN-based ferroelectric memristors as dynamic CMOS-compatible building blocks for in-memory and neuromorphic computing.

## 1. Introduction

With the continued advancement of artificial intelligence and electronic information technologies, there is a growing demand for computing systems that offer high speed and energy efficiency. In response, neuromorphic computing architectures that emulate the brain’s parallel and event-driven processing are gaining traction as alternatives to conventional von Neumann systems, which suffer from data transfer bottlenecks between memory and logic units [1,2]. Although synaptic behavior can be implemented using standard semiconductor devices, challenges such as high power consumption, limited scalability, and complex fabrication still hinder their widespread adoption. Therefore, device-level neuromorphic systems that can directly combine memory, nonlinear dynamics, and temporal information processing have become increasingly important.

To overcome these limitations, various emerging memory devices such as resistive memory, phase change memory [3], magnetic memory [4], and ferroelectric memory [5,6] are being explored for in-memory and neuromorphic computing. These devices can emulate synaptic functions through history-dependent conductance modulation and are also promising for physical reservoir computing because their intrinsic nonlinear and transient responses can process temporal input signals. However, many reported memristive and ferroelectric reservoir systems still face challenges such as device-to-device variability, cycle-to-cycle instability, stochastic switching, wake-up/fatigue effects, and insufficient control of volatile short-term dynamics [3,4,5,6]. Among them, ferroelectric memristors have attracted considerable attention because polarization switching can modulate the device conductance in a nonvolatile or volatile manner, enabling synaptic weight updates, short-term plasticity, and reservoir-state generation. Aluminum scandium nitride (AlScN), a wurtzite nitride ferroelectric, has recently emerged as a promising material owing to its strong spontaneous polarization, fast polarization switching, high thermal stability, and relatively wake-up-free behavior compared with hafnium-based ferroelectrics. Moreover, AlScN can be deposited by sputtering at low temperature and is compatible with back-end-of-line processes, making it attractive for integration with silicon electronics [7,8,9,10]. Despite these advantages, most AlScN-based studies have focused on ferroelectric memory, FeFETs, piezoelectric devices, or nonvolatile switching characteristics. The use of AlScN-based ferroelectric memristors as physical reservoirs that exploit volatile short-term memory for temporal information processing remains insufficiently explored.

To address this gap, we present a ferroelectric memristor based on a metal ferroelectric semiconductor (MFS) structure composed of TiN, AlScN, and heavily doped n^+^ silicon. The entire device stack was fabricated using low-temperature sputtering, ensuring compatibility with CMOS fabrication flows. Structural and electrical characterizations confirmed the formation of a high quality AlScN thin film with robust ferroelectric properties. The resulting device exhibited a high remanent polarization of approximately 80.91 μC/cm^2^ and maintained ferroelectric switching up to 10^5^ cycles under present measurement conditions. Beyond nonvolatile memory behavior, the device exhibited essential synaptic functions, including analog conductance modulation and short-term plasticity effects such as paired-pulse facilitation and depression. Importantly, the transient relaxation of conductance, associated with internal depolarization fields, enabled the emulation of short-term memory. This is a temporal feature extraction in reservoir computing [11,12,13,14]. Leveraging this property, we applied the device as a dynamic node in a reservoir computing framework using temporally encoded inputs, achieving a classification accuracy of 97.64% on the MNIST dataset. These findings suggest that the proposed silicon-compatible AlScN-based ferroelectric memristor can serve as a compact biomimetic physical reservoir platform by emulating biological synaptic functions, including gradual weight modulation, short-term plasticity, and short-term memory, for neuromorphic and in-memory computing. A direct comparison with representative AlScN-based devices is provided in Supplementary Table S1, highlighting the novelty of this work in terms of device type, device structure, remanent polarization, retention or memory behavior, energy consumption, synaptic function, and reservoir computing application.

## 2. Experiments

To fabricate an MFS structured device, deposition processes were conducted on a heavily doped n^+^Si substrate. The ferroelectric layer, AlScN, and the top electrode, TiN, were deposited via RF and direct current (DC) sputtering techniques, respectively. All sputtering processes were performed under high vacuum conditions with a base pressure maintained at 10^−6^ Torr to minimize contamination. The AlScN layer was deposited using an AlSc target containing 40 at. % scandium under an N_2_ flow of 20 sccm, applying an RF power of 300 W. For the TiN top electrode, patterning was first conducted, followed by DC sputtering at 150 W in an atmosphere of Ar (19 sccm) and N_2_ (1 sccm). The electrical characteristics of the fabricated single-cell device, with a 100 μm × 100 μm functional area, were evaluated using a Keithley 4200-SCS system equipped with a 4225-PMU module for pulse measurements under various conditions. PUND measurements and 2P_r_ uniformity analysis were conducted using 10 randomly selected cells. For DC I–V characterization, cells across the fabricated chip substrate were first electrically screened, and 10 representative functional cells from different sample regions were selected for detailed analysis. Synaptic conductance modulation, PPF/PPD, and reservoir computing pulse-response measurements were repeated using 10 functional cells under identical measurement conditions.

Additionally, the device structure was analyzed using cross-sectional high-resolution transmission electron microscopy (HRTEM). The HRTEM specimen was prepared by a standard focused ion beam (FIB) lift-out method, and a lamella containing the TiN/AlScN/n^+^ Si device stack was thinned for observation. The crystalline structure of the AlScN layer was further examined using grazing-incidence X-ray diffraction (GIXRD).

## 3. Results and Discussion

**Figure 1:**
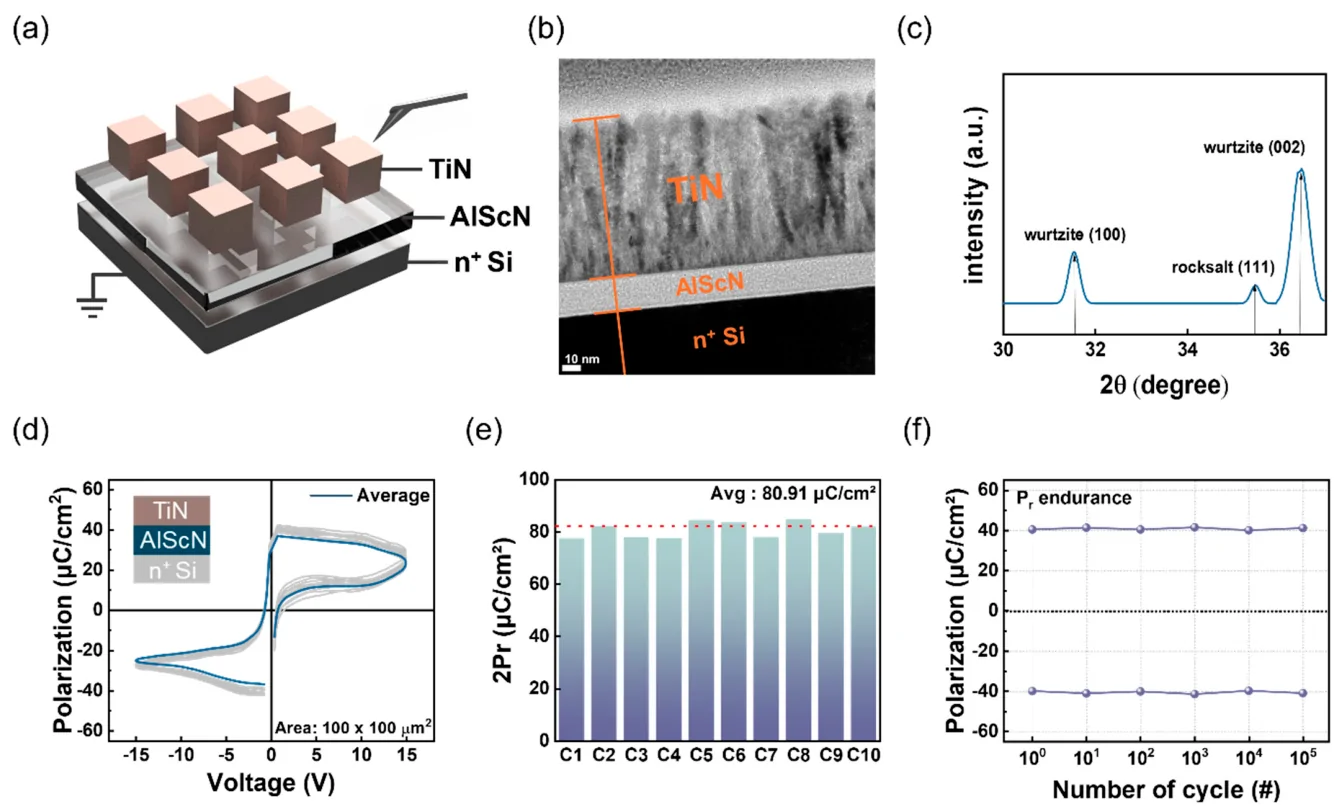
(**a**) The schematic illustration of the TiN/AlScN/n^+^poly Si ferroelectric memristor. (**b**) Cross-sectional transmission electron microscopy (TEM) image. (**c**) GIXRD spectrum of TiN/AlScN/n^+^Si. (**d**) Polarization-voltage curve of the device. (**e**) Cell-to-cell uniformity of 2P_r_ for 10 cells in the device. (**f**) Endurance test of the device up to 10^5^ cycles.

Figure 1a shows the schematic of the ferroelectric memristor fabricated through the described process. Each memory cell has lateral dimensions of 100 μm × 100 μm. Figure 1b displays a cross-sectional TEM image, confirming the successful formation of a 30 nm thick AlScN ferroelectric layer and 100 nm thick TiN electrodes. To investigate the chemical composition, energy-dispersive X-ray spectroscopy (EDS) was conducted, with the corresponding elemental mapping presented in Figure S1. The GIXRD pattern shown in Figure 1c verifies the formation of the wurtzite AlScN phase, as evidenced by a pronounced (002) peak and a relatively weak (100) reflection. The absence of a noticeable rock-salt (111) peak further indicates that the AlScN film predominantly grew with a c-axis-preferred orientation on the underlying stack. The ferroelectric properties were characterized using electrical measurements based on the positive-up-negative-down (PUND) method to extract the remnant polarization (2P_r_). In this approach, two identical positive pulses (P and U) and two identical negative pulses (N and D) are sequentially applied: the P and N pulses align the polarization, while the U and D pulses capture the non-switching current [15,16]. By subtracting the non-switching component, the actual ferroelectric switching current is isolated. Triangular voltage pulses with an amplitude of 15 V and a frequency of 500 kHz were applied. Figure 1d presents the PUND measurement results from 10 randomly selected cells, from which the average 2P_r_ was calculated. The individual hysteresis loops are shown in Figure S2. The polarization-voltage loop measured within the safe voltage window exhibits incomplete closure and a narrowed apparent coercive field. This non-ideal behavior can be attributed to two possible factors. First, residual oxygen- or moisture-related impurities in the sputtering plasma may generate defect states or interfacial oxide regions, thereby increasing leakage-assisted charge transport and partial domain pinning. These effects can disturb the local electric-field distribution across the AlScN layer and broaden the distribution of local switching thresholds. Second, because the PUND measurement was performed within a safe voltage window to avoid dielectric breakdown, some strongly pinned domains may remain incompletely switched. As a result, only partially switched polarization states are obtained, leading to incomplete loop closure and an apparently narrowed coercive field. Therefore, the narrowed coercive field should be regarded as an apparent non-ideal response rather than an intrinsic reduction in the coercive field of AlScN [17,18,19,20].

These observations motivate targeted process optimization in our follow-up study. Refabrication using ultra-high-purity N_2_/Ar gases and the introduction of oxygen-inert electrodes or interfacial barrier layers will be considered to suppress oxygen- or moisture-related defect formation, reduce leakage current, alleviate domain pinning, and enable fully saturated P-V loops.

As summarized in Figure 1e, the average 2P_r_ value was 80.91 μC/cm^2^, indicating excellent uniformity in ferroelectric switching across cells. AlScN-based ferroelectric devices are known to exhibit no wake-up effect, in contrast to hafnium-based counterparts [21,22]. To evaluate device reliability, cycling endurance tests were conducted. As shown in Figure 1f, the device maintained stable ferroelectric switching behavior up to 10^5^ cycles under the present measurement conditions. Although this endurance level supports the feasibility of the device for neuromorphic and reservoir computing demonstrations, it remains lower than the endurance typically required for competitive nonvolatile ferroelectric memory technologies. Therefore, further optimization of the AlScN deposition process, electrode/interface engineering, and leakage suppression will be necessary to improve cycling reliability for practical large-scale in-memory computing applications [23,24].

**Figure 2:**
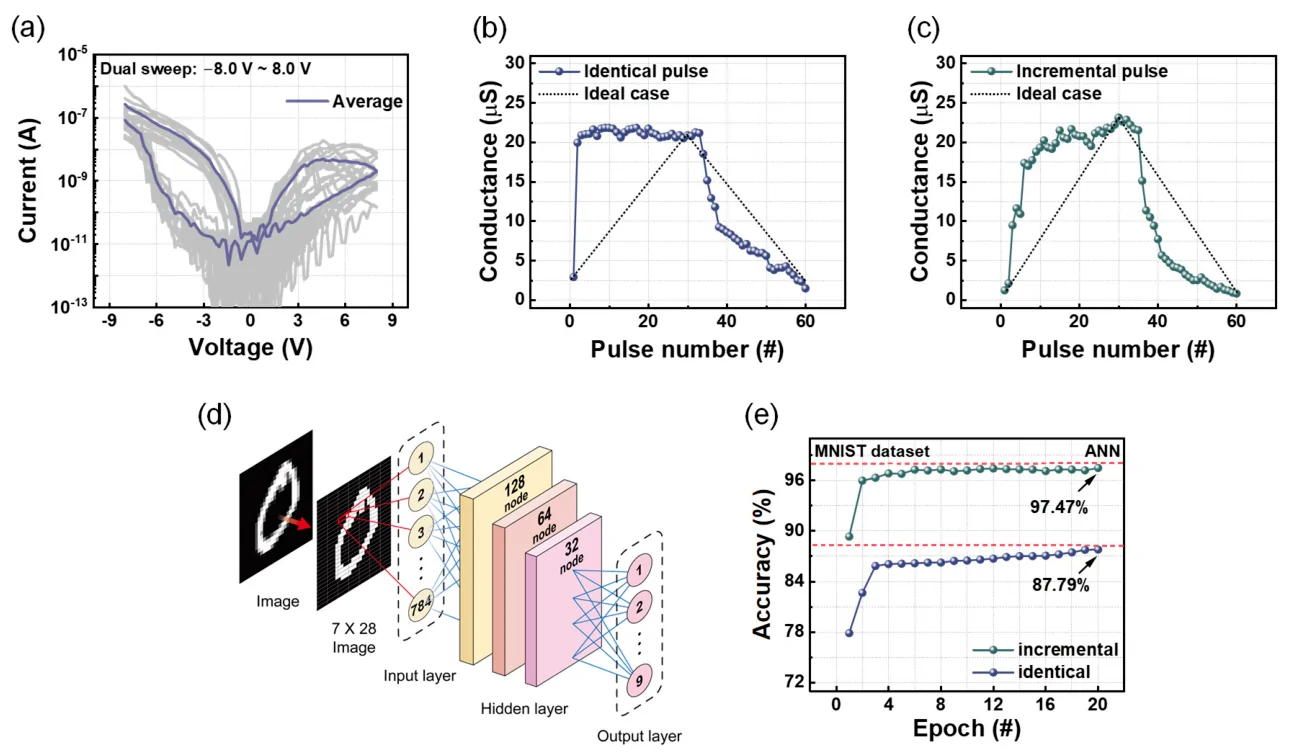
(**a**) Cell-to-cell I–V characteristics of 10 cells. Potentiation and depression in (**b**) identical and (**c**) incremental pulse schemes. (**d**) Configuration of a neural network applied to MNIST dataset. (**e**) Comparison of pattern recognition accuracy on the MNIST dataset using identical and incremental pulses.

As shown in Figure 2a, the electrical characteristics of the fabricated device were evaluated through DC voltage sweeps from −8 V to +8 V with 0.1 V increments. To assess the array-level reliability, cells across the entire chip substrate were electrically screened. A cell was classified as functional when it exhibited measurable memristive behavior without short/open failure or hard breakdown during the voltage sweep. Based on this criterion, the overall device yield across the fabricated chip substrate was approximately 80%. In addition, ten representative functional cells located in different regions of the memristor sample were selected to examine the spatial consistency of the I–V characteristics; the detailed results are provided in Figure S3. All ten representative cells exhibited stable and reproducible I–V curves, indicating that consistent memristive behavior was observed across different regions of the sample. Synaptic behaviors were further analyzed through conductance modulation experiments, focusing on potentiation (conductance increase) and depression (conductance decrease) [25,26]. The identical pulse scheme, illustrated in Figure 2b, consisted of applying a train of 30 identical set pulses at 10 V for potentiation and 30 identical reset pulses at −3 V for depression, each followed by a 4 V read pulse. All pulses had a uniform duration of 300 μs. To improve the linearity and symmetry of the conductance change, an incremental pulse approach was adopted (Figure 2c), wherein 30 set pulses gradually increasing from 8 V to 10 V and 30 reset pulses gradually decreasing from −2.1 V to −3.1 V were applied, maintaining a consistent pulse width of 300 μs. Both methods demonstrated gradual changes in conductance; however, the incremental pulse approach resulted in more linear and uniform modulation compared to the identical pulse method. The improved linearity obtained with the incremental pulse scheme can be understood in terms of field-dependent polarization switching and gradual modulation of the interfacial potential barrier. Under identical pulses, low-threshold domains or charge states are preferentially switched during the early pulses, leading to a large initial conductance change followed by saturation. This results in nonlinear potentiation and depression behavior. In contrast, the incremental pulse scheme gradually increases the effective electric field during potentiation and depression, enabling domains or interfacial states with different switching thresholds to be progressively activated. Therefore, conductance modulation is distributed more uniformly over the pulse sequence, suppressing abrupt switching and improving the linearity of synaptic weight updates [27,28].

To explore the applicability of the device in neuromorphic computing, an online learning simulation was performed using the MNIST dataset for digit recognition. Figure 2d illustrates the employed artificial neural network (ANN), which comprised an input layer with 784 nodes (corresponding to 28 × 28 pixel images), three hidden layers containing 128, 64, and 32 neurons respectively, and a 10-neuron output layer corresponding to the ten-digit classes. The experimentally measured conductance modulation characteristics obtained under the identical and incremental pulse schemes were used to emulate synaptic weight update behavior [29,30]. Details of the MNIST input representation, ANN forward propagation, and conductance-based weight update mapping are provided in Supplementary Note S1.

After 20 epochs, the ANN achieved recognition accuracies of 97.47% with the incremental pulse scheme and 87.79% with the identical pulse scheme, indicating a significant improvement (9.68%) through the incremental method (Figure 2e). Confusion matrices for both methods are presented in Figure S4.

**Figure 3:**
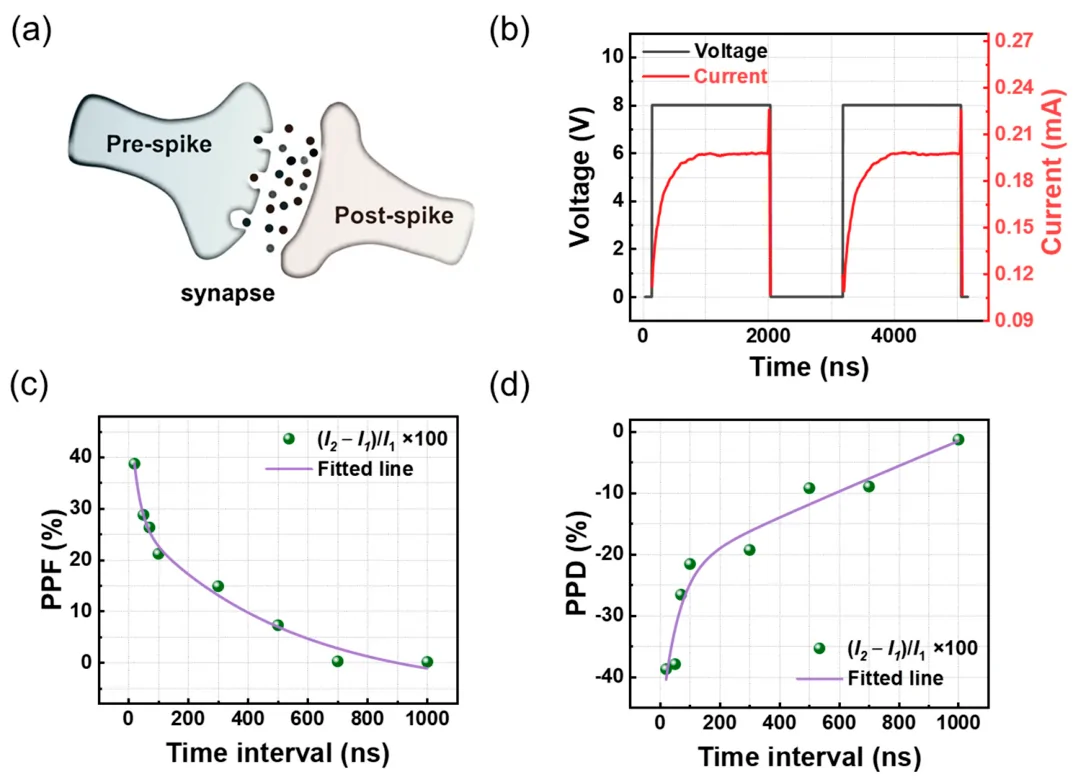
(**a**) Illustration of a biological neural network. (**b**) PPF characteristics of the device. (**c**) PPF index over time interval. (**d**) PPD index over time interval.

As illustrated in Figure 3a, the ferroelectric layer connecting the top and bottom electrodes can function as an artificial synapse, where n^+^ Si and TiN electrodes correspond to the pre-synaptic and post-synaptic neurons, respectively [31]. In neuroscience, paired-pulse facilitation (PPF) and paired-pulse depression (PPD) represent critical short-term synaptic plasticity mechanisms. These phenomena occur when two identical stimuli are sequentially applied with a defined interval, allowing the initial stimulus to influence the response to the subsequent one. Specifically, PPF results in an enhanced excitatory post-synaptic current (EPSC) when the interval between pulses is short, whereas PPD leads to a reduced inhibitory post-synaptic current (IPSC) under similar conditions [32,33,34]. To emulate these synaptic behaviors, we experimentally evaluated both PPF and PPD characteristics. Figure 3b shows the current responses to two identical voltage pulses (8 V, 2 μs) separated by a 1 μs interval; positive pulses were applied for PPF, and negative pulses (−8 V, 2 μs) for PPD. Figure 3c,d illustrate how the PPF and PPD indices vary with pulse intervals ranging from 20 ns to 1 μs. These measurements were designed to evaluate fast volatile paired-pulse plasticity under short inter-pulse intervals, thereby capturing the transient facilitation and depression behavior associated with the STM characteristics of the AlScN-based ferroelectric memristor. The PPF/PPD index was calculated using the following equation [35]:

(1) $$PPF\;index=\;\frac{\left(I_{2}-I_{1}\right)}{I_{1}}\;\times 100\;\;\;\;\;$$

where $I_{1}$ is the average current of the first pulse, and $I_{2}$ represents that of the second.

The measured PPF values were fitted using a double exponential decay model expressed as Equation (2) [36]:

(2) $$y\;=\;y_{0}+A_{1}e^{-\frac{x}{t_{1}}}+A_{1}e^{-\frac{x}{t_{2}}}\;\left(t_{i}>0,i=1,2\right)\;\;\;\;\;$$

where $y_{0}$ is a constant baseline, *A*_1_ and *A*_2_ indicate the initial amplitude components, and $t_{1}$ and $t_{2}$ correspond to characteristic relaxation times. As illustrated in Figure 3c,d, the PPF and PPD indices exhibit exponential trends with decreasing pulse intervals, showing an increase in PPF and a decrease in PPD. These trends are consistent with biological synaptic behavior. These results confirm that the proposed device effectively emulates short-term synaptic dynamics, highlighting its strong potential for integration into neuromorphic systems.

The STM behavior observed in the AlScN-based MFS memristor can be attributed to partial polarization relaxation driven by the internal depolarization field. Based on a series-capacitor approximation, the depolarization field was estimated to be approximately 3.5 MV cm^−1^, as described in Supplementary Note S2. This internal field can assist the relaxation of the polarization-induced conductance state after removal of the external pulse, thereby contributing to the observed volatile STM behavior [37].

**Figure 4:**
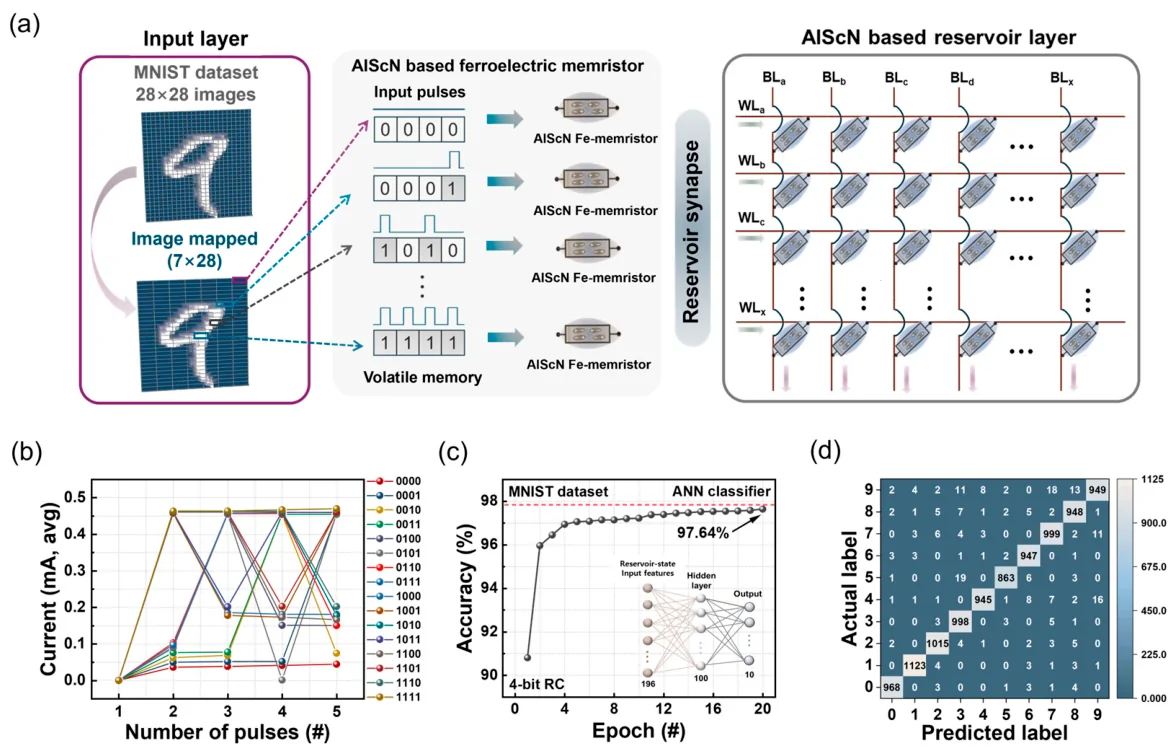
(**a**) Schematic of reservoir computing structure. (**b**) Device reactions to 16 different 4-bit input pulse sequences. (**c**) MNIST dataset recognition accuracy using reservoir computing. (**d**) Confusion matrix derived from inference results on pattern testing.

Finally, we implemented reservoir computing (RC) by utilizing the STM characteristics of the AlScN-based ferroelectric memristor, enabling the device to serve as a physical reservoir for processing temporally varying inputs [38]. As illustrated in Figure 4a, the RC workflow consists of temporal input encoding, transformation of the input signals by the AlScN physical reservoir, and subsequent software-based classification using the extracted reservoir states. To perform digit recognition using the MNIST dataset, the original 28 × 28 grayscale images were first binarized: pixels with values between 0 and 127 were assigned 0 (black), whereas those between 128 and 255 were assigned 1 (white). The resulting 784 binary pixels were then grouped into sets of four, yielding 196 four-bit input sequences for each image. Each four-bit sequence was represented as a temporal pulse train and mapped to the experimentally measured response of the AlScN memristor reservoir [39,40,41]. As shown in Figure 4b, the 16 possible four-bit patterns, ranging from 0000 to 1111, were represented using 8 V pulses for bit “1”, 0 V for bit “0”, and a 4 V read pulse. All pulses had a duration of 100 μs. This pulse width used for the 4-bit RC patterns was selected as an input-encoding condition to obtain stable and distinguishable current responses for the 16 possible four-bit input patterns. Under this pulse scheme, the experimentally measured pattern-dependent current responses were used as reservoir-state features for the offline RC simulation. Specifically, the current responses of the AlScN memristor to the 16 possible four-bit pulse patterns were first measured experimentally, and the corresponding values are provided in Figure S5. The RC simulation was performed as a device-informed offline simulation based on experimentally measured device responses. These measured responses were then used as a reservoir-state mapping table. During the offline MNIST simulation, each four-bit input sequence was converted into the corresponding experimentally measured reservoir-state current feature, resulting in a 196-dimensional feature vector for each image. The extracted 196 reservoir-state features were then fed into a software ANN classifier consisting of 196 input neurons, 100 hidden neurons, and 10 output neurons corresponding to the ten-digit classes [42,43]. Thus, the physical AlScN memristor functioned as the reservoir for temporal feature extraction, while the subsequent classification was performed offline in software. The RC simulation achieved a recognition accuracy of 97.64% after 20 training epochs, as shown in Figure 4c, demonstrating the potential of the AlScN-based ferroelectric memristor for neuromorphic pattern recognition. The confusion matrix for the classification results is presented in Figure 4d. The strong diagonal distribution indicates that most MNIST digits were correctly classified using the reservoir-state features extracted from the AlScN memristor. The remaining errors were mainly observed between digits with similar handwritten shapes, such as 4 and 9, 7 and 9, and 3 and 5, suggesting that the misclassifications primarily originate from intrinsic similarities in the input digit patterns. These results confirm that the AlScN-based ferroelectric memristor can effectively provide distinguishable reservoir-state features for neuromorphic pattern recognition.

## 4. Conclusions

In conclusion, we successfully fabricated and characterized the MFS structured memristor based on AlScN ferroelectric thin films, highlighting its significant potential for neuromorphic and in-memory computing applications. The RF-sputtered AlScN layer exhibited a wurtzite crystal phase with a dominant c-axis-preferred orientation, as confirmed by the GIXRD pattern. Electrical characterization using the PUND method revealed a high remanent polarization (2P_r_) of 80.91 μC/cm^2^ and good cell-to-cell uniformity. The device also maintained stable ferroelectric switching up to 10^5^ cycles under the present measurement conditions, supporting its feasibility for neuromorphic and reservoir computing demonstrations while indicating that further endurance improvement is required for practical nonvolatile memory applications. The device demonstrated stable and symmetric conductance modulation using both identical and incremental pulse methods. Notably, the incremental pulse approach enhanced linearity and precision in synaptic weight updates, resulting in a 9.68% improvement in MNIST digit recognition accuracy. Additionally, the device successfully emulated short-term synaptic plasticity characteristics such as PPF and PPD, closely matching biological synaptic behavior. Leveraging its short-term memory capability, the AlScN-based memristor achieved an impressive classification accuracy of 97.64% on the binarized MNIST dataset within a reservoir computing framework, underscoring its effectiveness as a physical reservoir for temporal data processing.

## Data Availability

The data that support the findings of this study are available within the article and its Supplementary Material.

## Supplementary Materials

The following supporting information can be downloaded at: https://www.mdpi.com/article/10.3390/biomimetics11080519/s1, Figure S1: Cross-sectional TEM–EDS analysis of the TiN/AlScN/n^+^ Si device stack; Figure S2: PUND measurement results for 10 cells; Figure S3: I–V measurements for 10 cells; Figure S4: Confusion matrices obtained from pattern-testing inference using the identical and incremental pulse schemes; Figure S5: Representation of 16 states using 4-bit binary codes; Table S1: Comparison of representative AlScN-based studies from the perspective of biomimetic neuromorphic processes; Note S1: MNIST-based online learning simulation; Note S2: Estimation of the depolarization field in the AlScN-based MFS memristor. References [44,45,46,47,48,49] are cited in the Supplementary Materials.
