# Supplementary material for: CMOS-Compatible AlScN Memristor on Silicon Exhibiting Short-Term Memory for Reservoir Computing
Source: Biomimetics (Basel). 2026 Jul 23;11(8):519. doi: 10.3390/biomimetics11080519 (PMC13510034; doi:10.3390/biomimetics11080519)
Supplement: Supplementary file 1 [file biomimetics-11-00519-s001.zip › biomimetics-4406680-supplementary.pdf]

Supplementary information

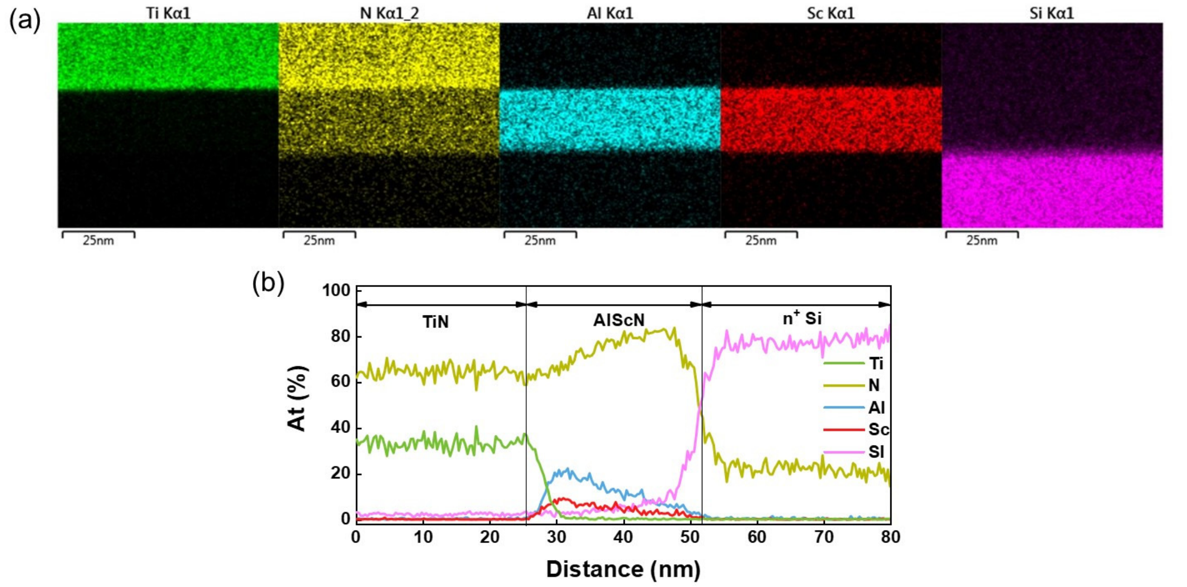

Figure S1. Cross-sectional TEM–EDS analysis of the TiN/AlScN/n^{+} Si device stack. (a) EDS elemental mapping images showing the spatial distribution of the constituent elements. (b) EDS line-scan profile obtained across the TiN/AlScN/n^{+} Si stack.

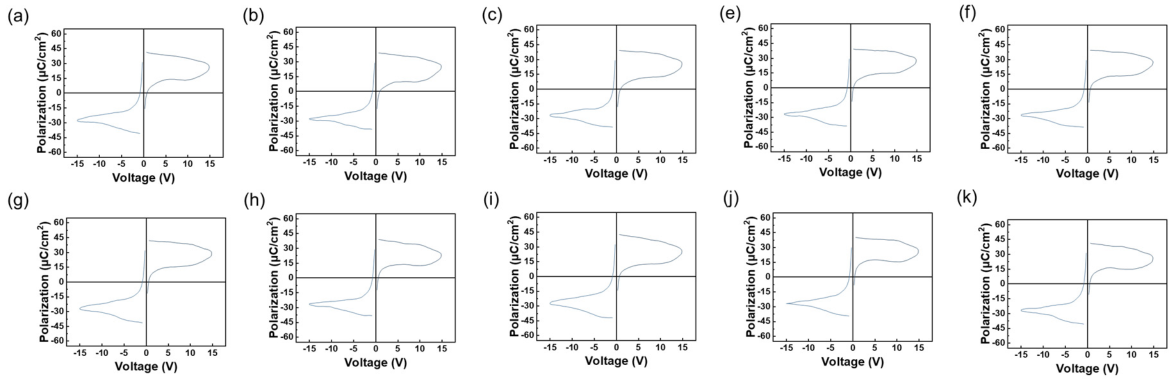

Figure S2. (a-k) PUND measurement results for 10 cells of the device

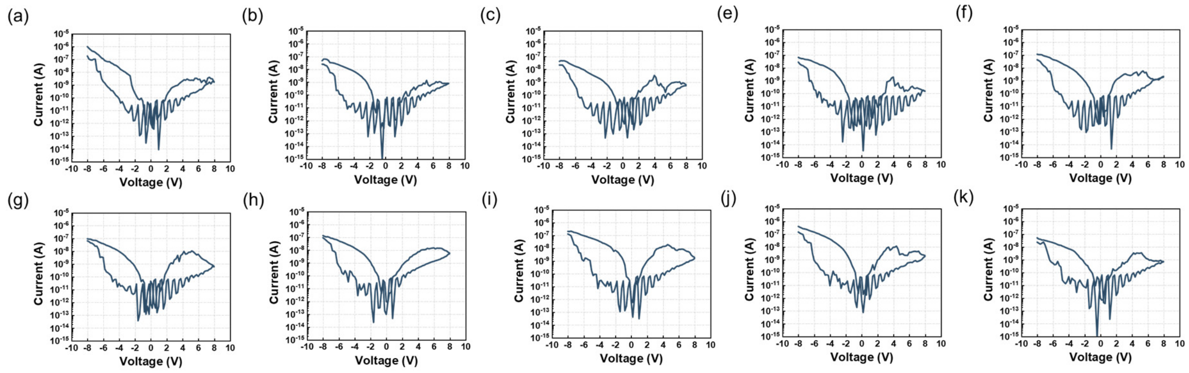

Figure S3. IV measurements for 10 cells of the device

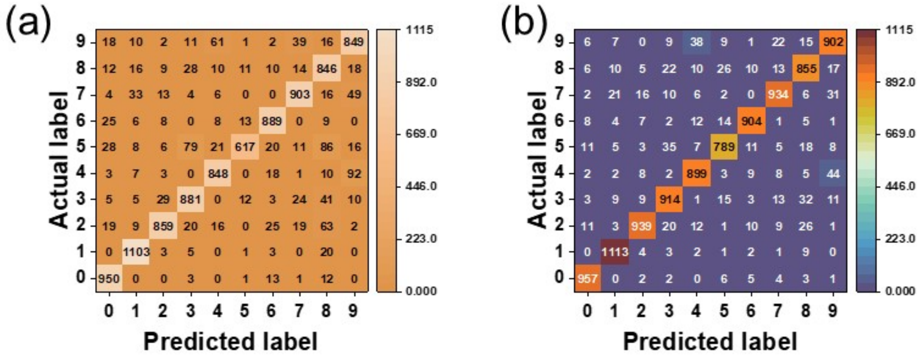

Figure S4. Confusion matrices obtained from inference results during pattern testing using (a) identical pulse scheme and (b) incremental pulse scheme

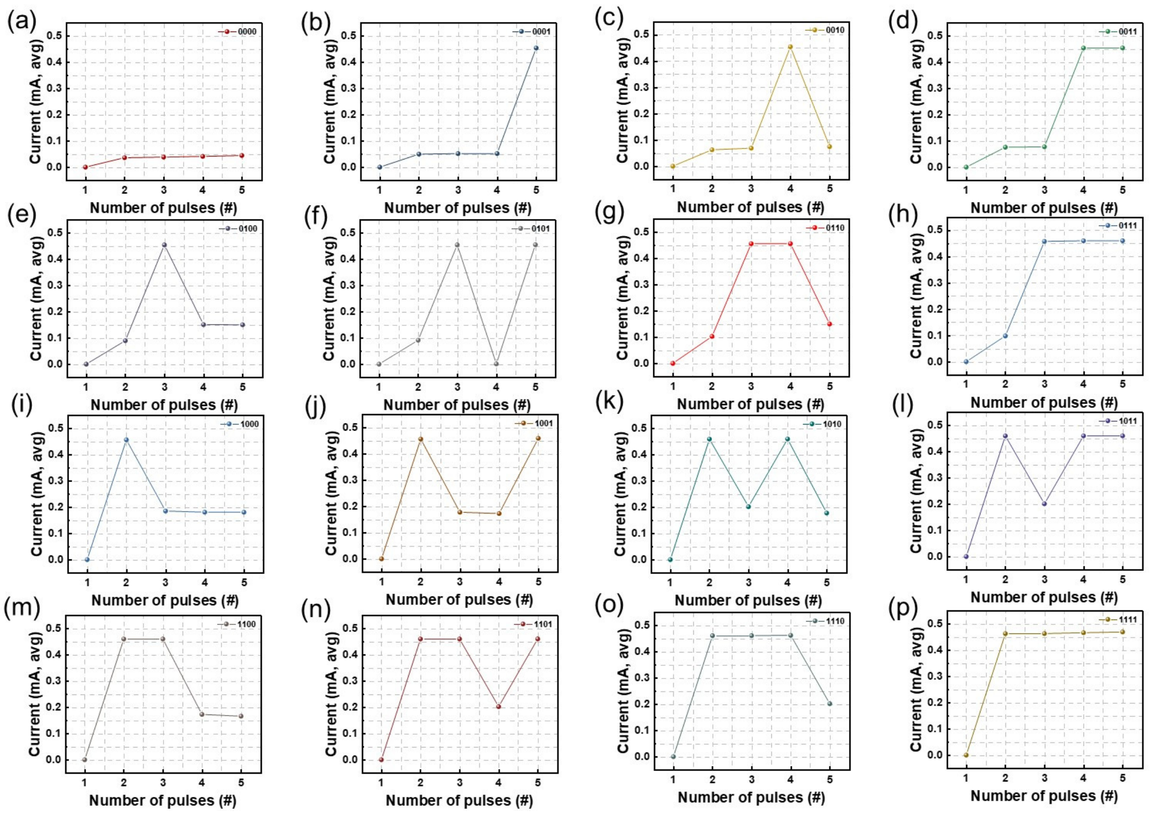

Figure S5. 16 states represented using 4 bits. Each state is tested on average three times, with all measurements obtained from a single cell. The binary numbers corresponding to these states are: (a) 0000, (b) 0001, (c) 0010, (d) 0011, (e) 0100, (f) 0101, (g) 0110, (h) 0111, (i) 1000, (j) 1001, (k) 1010, (l) 1011, (m) 1100, (n) 1101, (o) 1110, and (p) 1111.

Table S1. Comparison of representative AlScN-based studies from the perspective of biomimetic neuromorphic processes.

Type	Device Structure	Polarization (μC cm^{-2})	Retention	Energy consumption	Synaptic Function	Application	Ref.
Capacitor	Pt/$Al_{0.64}Sc_{0.36}N$/Pt	~100	LTM	0.93 M/V	-	-	[1]
FeFET	AlScN/MoS_2 (MFMIS)	104	LTM	-	Conductance modulation, LTP/LTD	Image classification	[2]
Memristor	Cu/$Al_{0.73}Sc_{0.27}N$/Pt	-	LTM/STM	21.4 pJ	Conductance modulation, STP-to-LTP, PPF	-	[3]
Memristor	ITO/AlScN/n^+poly Si	48.46	STM	0.283 mW/cm^{-2}	Conductance modulation, EPSC, STP/STD	Reservoir computing , Image classification	[4]
Memristor	TiN/AlScN/n^+Si	45.45	STM	3.5 M/V	Conductance modulation, PPF/PPD STP/STD	Reservoir computing , Image classification/ recognition	This Work

Note S1. MNIST-based online learning simulation

To clarify the MNIST-based online learning simulation used in Figure 2(d), the input representation, ANN architecture, and conductance-based weight update mapping are described in detail. Each MNIST image was treated as a 28 x 28 grayscale image and converted into a 784-dimensional input vector,

$$x = [x_1, x_2, \dots, x_{784}]^T,$$

where x_i represents the intensity of the i-th pixel. This 784-dimensional vector was applied to the input layer of the ANN.

The ANN consisted of an input layer with 784 nodes, three hidden layers containing 128, 64, and 32 neurons, respectively, and a 10-neuron output layer corresponding to the ten digit classes. The forward propagation of the ANN can be expressed as [6]

$$a^{(l)} = f^{(l)}\left(W^{(l)}a^{(l-1)} + b^{(l)}\right),$$

where $a^{(l)}$, $W^{(l)}$, and $b^{(l)}$ represent the activation vector, weight matrix, and bias vector of the l-th layer, respectively, and $f^{(l)}(\cdot)$ denotes the activation function of the l-th layer, and $a^{(0)} = x$.

The experimentally measured conductance modulation characteristics of the AlScN-based ferroelectric memristor were used to emulate synaptic weight update behavior. The potentiation and depression characteristics measured under the identical and incremental pulse schemes were used to define the positive and negative weight update behaviors, respectively. During the online learning process, synaptic weights were updated sequentially during training. Positive weight updates were mapped to the measured potentiation behavior, whereas negative weight updates were mapped to the measured depression behavior.

The conductance-based weight update can be described as [7]

$$\Delta w^+ \propto \Delta G_P, \qquad \Delta w^- \propto \Delta G_D,$$

Where Δw^+ and Δw^- represent the positive and negative synaptic weight updates, respectively. ΔG_P and ΔG_D represent the conductance changes induced by potentiation and depression pulses, respectively. These conductance changes can be written as

$$\Delta G_P = G_{P,n+1} - G_{P,n} > 0,$$
$$\Delta G_D = G_{D,n+1} - G_{D,n} < 0,$$

Where $G_{P,n}$ and $G_{D,n}$ denote the conductance states after the n-th potentiation and depression pulse, respectively. Therefore, the simulation reflects how the experimentally measured conductance update behavior influences the ANN learning performance. The improved linearity of the incremental pulse scheme resulted in more gradual and symmetric weight updates, leading to higher MNIST recognition accuracy than the identical pulse scheme.

Note S2. Estimation of the depolarization field in the AlScN-based MFS memristor

The STM behavior observed in our AlScN-based MFS memristor can be attributed to partial polarization relaxation driven by the internal depolarization field (E_{dep}). This depolarization field originates from incompletely compensated bound charges at the AlScN layer and the interfacial dielectric layer as a series capacitor system, E_{dep} can be estimated as follows:

$$E_{dep} = -\frac{P_r t_d}{\varepsilon_0(\varepsilon_d t_f + \varepsilon_f t_d)} ,$$

where P_r is the remanent polarization, t_f and ϵ_f are the thickness and relative permittivity of the AlScN layer, respectively, and t_d and ϵ_d are the thickness and relative permittivity of the interfacial dielectric layer, respectively.

For the present TiN/AlScN/n^+Si stack, t_f was 30 nm, as confirmed by the cross-sectional TEM image. From the PUND measurement, $2P_r$ was 80.91 μC/cm^2, corresponding to P_r = 40.45 μC/cm^2. For the estimation, $\epsilon_f = 12$ was used as a representative relative permittivity of sputtered AlScN, while the interfacial layer was approximated as a thin native/interfacial oxide with $\epsilon_d = 3.9$ and t_d = 1 nm. Using these parameters, E_{dep} was estimated to be approximately 3.5 MV/cm.

This value is comparable to the apparent coercive field of the AlScN layer extracted from the P-V curve, suggesting that the internal depolarization field can assist partial polarization relaxation after removal of the external pulse. Therefore, the observed volatile STM behavior is attributed to depolarization-field-assisted partial polarization relaxation in the TiN/AlScN/n^+Si structure.

References

1. Fichtner, S.; Lofink, F.; Wagner, B.; Schönweger, G.; Kreutzer, T.-N.; Petraru, A.; Kohlstedt, H. Ferroelectricity in AlScN: Switching, Imprint and Sub-150 nm Films. In *Proceedings of the 2020 IEEE IFCS-ISAF*, Keystone, CO, USA, 19–23 July 2020; pp. 1–4. https://doi.org/10.1109/IFCS-ISAF41089.2020.9234883

2. Gao, J.; Chien, Y.-C.; Li, L.; Lee, H.K.; Samanta, S.; Varghese, B.; Xiang, H.; Li, M.; Liu, C.; Zhu, Y.; Chen, L.; Ang, K.-W. Ferroelectric Aluminum Scandium Nitride Transistors with Intrinsic Switching Characteristics and Artificial Synaptic Functions for Neuromorphic Computing. *Small* 2024, *20*, 2404711. https://doi.org/10.1002/smll.202404711

3. Han, C.; Wei, Y.; Qin, H.; Zhang, M.; Wang, Y.; Hu, R.; He, N.; Wu, Y.; Wu, B.; Zhang, H.; Wang, X.; Liu, Y.; Tong, Y. Artificial Synaptic Simulation Using Wurtzite AlScN-Based Ferroelectric Memristors. *ACS Appl. Electron. Mater.* 2025, *7*, 380–387. https://doi.org/10.1021/acsaelm.4c01566.

4. Park, W.; Chae, H.; Park, J.; Kim, S.; Park, C.; Seo, Y.; Kim, S. AlScN-Based Ferroelectric Memristor for Electrical Synapse Emulation and Light-Stimulated Reservoir Computing. *J. Chem. Phys.* **2025**, *163*, 234707. https://doi.org/10.1063/5.0298621

5. Goodfellow, I.; Bengio, Y.; Courville, A. *Deep Learning*; MIT Press: Cambridge, MA, USA, 2016.

6. Kim, S.; Lim, M.; Kim, Y.; Kim, H.-D.; Choi, S.-J. Impact of Synaptic Device Variations on Pattern Recognition Accuracy in a Hardware Neural Network. *Sci. Rep.* 2018, *8*, 2638.
